# HLA-Restriction of Human Treg Cells Is Not Required for Therapeutic Efficacy of Low-Dose IL-2 in Humanized Mice

**DOI:** 10.3389/fimmu.2021.630204

**Published:** 2021-02-24

**Authors:** Rajeev K. Tyagi, Justin Jacobse, Jing Li, Margret M. Allaman, Kevin L. Otipoby, Erik R. Sampson, Keith T. Wilson, Jeremy A. Goettel

**Affiliations:** ^1^ Division of Gastroenterology, Hepatology and Nutrition, Department of Medicine, Vanderbilt University Medical Center, Nashville, TN, United States; ^2^ Willem-Alexander Children’s Hospital, Department of Pediatrics, Leiden University Medical Center, Leiden, Netherlands; ^3^ Department of Pathology, Microbiology, and Immunology, Vanderbilt University Medical Center, Nashville, TN, United States; ^4^ Pandion Therapeutics, Immunology Department, Watertown, MA, United States; ^5^ Program in Cancer Biology, Vanderbilt University School of Medicine, Nashville, TN, United States; ^6^ Vanderbilt Institute for Infection, Immunology, and Inflammation, Vanderbilt University Medical Center, Nashville, TN, United States; ^7^ Center for Mucosal Inflammation and Cancer, Vanderbilt University Medical Center, Nashville, TN, United States; ^8^ Veterans Affairs Tennessee Valley Healthcare System, Nashville, TN, United States

**Keywords:** IBD, IL-2, humanized, colitis, Tregs

## Abstract

Regulatory T (T_reg_) cells are essential to maintain immune homeostasis in the intestine and T_reg_ cell dysfunction is associated with several inflammatory and autoimmune disorders including inflammatory bowel disease (IBD). Efforts using low-dose (LD) interleukin-2 (IL-2) to expand autologous T_reg_ cells show therapeutic efficacy for several inflammatory conditions. Whether LD IL-2 is an effective strategy for treating patients with IBD is unknown. Recently, we demonstrated that LD IL-2 was protective against experimental colitis in immune humanized mice in which human CD4^+^ T cells were restricted to human leukocyte antigen (HLA). Whether HLA restriction is required for human T_reg_ cells to ameliorate colitis following LD IL-2 therapy has not been demonstrated. Here, we show that treatment with LD IL-2 reduced 2,4,6-trinitrobenzensulfonic acid (TNBS) colitis severity in NOD.*Prkdc^scid^Il2rg^-/-^* (NSG) mice reconstituted with human CD34^+^ hematopoietic stem cells. These data demonstrate the utility of standard immune humanized NSG mice as a pre-clinical model system to evaluate therapeutics targeting human T_reg_ cells to treat IBD.

## Introduction

IBD is a chronic relapsing and remitting disorder broadly classified as either Crohn’s disease (CD) or ulcerative colitis (UC) (1–3). UC is an idiopathic disease of the large intestine with continuous lesions, whereas CD may occur anywhere along the gastrointestinal tract and is characterized by discontinuous lesions often with transmural inflammation. Over 1.4 million individuals are afflicted with IBD in the United States alone, carrying an annual economic burden of nearly $30 billion and an estimated 6–8 million patients world-wide (4–7). The pathogenesis of IBD is multifactorial and driven by immunological, environmental, genetic, and microbial factors (8–10). Dysfunctional regulatory mechanisms of the immune system can trigger disease onset or exacerbate IBD. In recent years, genome-wide association studies have identified polymorphisms that confer increased risk for IBD with many occurring to genes involved in immune regulation or host defense (9, 10). Nevertheless, the molecular mechanisms driving IBD are not fully understood (10–13).

While there is no cure for IBD, current strategies to induce remission include immunosuppression or biologics that neutralize pro-inflammatory cytokines or impede immune cell trafficking (14–16). Though many patients initially respond to these therapeutic approaches, long-term immune suppression may increase the risk of infection and many patients that do respond become refractory to treatment. Recent efforts to selectively augment the number or function of T_reg_ cells to treat chronic inflammatory diseases have yielded promising results (17). Work from Desreumaux and colleagues shows that following the transfer of *ex vivo* expanded autologous antigen-specific T_reg_ cells is effective in treating refractory CD (18). However, access to good manufacturing practice-compliant facilities may limit broad implementation of such approaches. It has been appreciated for some time that T_reg_ cells constitutively express CD25, a component of the trimeric IL-2 receptor complex, which increases the affinity of the receptor complex for IL-2 (19). This property enables T_reg_ cells to bind IL-2 at lower concentrations compared to conventional T cells. Recent work has documented the efficacy of LD IL-2 in the treatment of various chronic inflammatory disorders including graft-versus-host disease, systemic lupus erythematosus, Wiskott-Aldrich syndrome, and hepatitis C virus-induced vasculitis due to the selective expansion of T_reg_ cells (20–23).

Logistical and ethical considerations involving human subject research, combined with biological limitations of cell culture, make murine models attractive surrogates to study human diseases (24). However, investigations pertaining to immune regulation may limit their utility when evaluating human therapeutics or drug targets (25). To overcome this limitation, we and others have employed immunodeficient NOD.*Prkdc^scid^.Il2rg^-/-^* (NSG) mice, or similar strains, that undergo near complete human hematopoiesis when injected at birth with human CD34^+^ hematopoietic stem cells (HSCs) (26–34). However, despite robust lymphocyte development in these human immune system (HIS)-mice, adaptive immune responses are notoriously sub optimal due to thymic selection of human T cells being restricted to murine major histocompatibility complex (MHC) class I/II molecules. Previously we developed NSG mice deficient for MHCII and in which human CD4 T cells were selected and restricted to human leukocyte antigen (HLA). Using these mice, we established that HLA-restriction was required to phenocopy a human disease due to the patient’s monogenic immune disorder (26). We then employed HLA-restricted humanized mice as a pre-clinical model to assess the efficacy of LD IL-2 in IBD and found that LD IL-2 expanded human T_reg_ cells and was protective against experimental colitis (28). Nevertheless, it is unclear if LD IL-2 T_reg_ cell expansion and suppression of effector responses requires T_reg_ cell T cell receptor-HLA interactions. Here, we report that the therapeutic effects of LD IL-2 is independent of T_reg_ cell HLA-restriction and demonstrate the utility of immune humanized NSG mice as a platform to evaluate therapeutics targeting human T_reg_ cells.

## Methods

### Humanized Mice

NOD.*Prkdc^scid^Il2rg^m1Wjl^/SzJ* (NSG) mice reconstituted with human CD34^+^ hematopoietic stem cells (HSCs) showing more than 25% human CD45^+^ cells in peripheral blood were procured from The Jackson Laboratory (Bar Harbor, ME, USA). HSCs from four unique donors were used to reconstitute mice. All mice were maintained in the specific-pathogen free facility and animal experiments were approved by the Institutional Animal Care and Use Committee (IACUC) of Vanderbilt University Medical Center, Nashville, TN, USA.

### Immunophenotypic Characterization and Antibodies

Immune cells were isolated from blood and spleens of NSG humanized mice at the experimental endpoint. Single cell suspensions of mouse blood and spleen underwent red blood cell lysis using ACK lysis buffer (ThermoFisher, Waltham, MA, USA) followed by passage through 70 μm cell strainer (BD Biosciences, San Jose, CA, USA). Immune cells were first blocked in 10% rat serum, followed by the incubation with fluorochrome-conjugated antibodies for 15 min at 4°C in PBS and washed with PBS containing 2% fetal calf serum (FACS buffer). Antibodies used for immunophenotyping: CD3, clone SK7 and CD8, clone RPA-T8 were purchased from eBioscience (San Diego, CA, USA). CD25, clone M-A251; CD45, clone HI30; CD127, clone A019D5; ICOS, clone C398.4A; CTLA4, clone BNI3; CD45RO, clone UCHL1; CD69, clone FN50; HLA-DR, clone L243; CD45RA, clone HI100; CD16, clone 3G8 and FOXP3, clone 206D were purchased from BioLegend (San Diego, CA, USA). CD4, clone RPA-T4 antibody was purchased from BD Biosciences (San Jose, CA, USA). Live/Dead fixable aqua dead cell stain kit was purchased from Thermo Fisher. Intracellular FOXP3 staining was performed by employing the eBioscience™ FOXP3/Transcription Factor Staining Buffer Set according to the manufacturer’s protocol. The anti-FOXP3 monoclonal antibody (clone 206D) was purchased from eBioscience. Samples were acquired using a LSRFortessa flow cytometer (BD Biosciences) and data were analyzed using FlowJo Software (Tree Star, Inc., Ashland, OR, USA).

### Low-Dose IL-2 Treatment

IL-2 (Proleukin™, Prometheus Laboratories Inc., San Diego, CA, USA) was reconstituted in PBS and mice were administered daily injections of 10,000 IU starting at the time of sensitization and continued until the experimental endpoint.

### Induction of Colitis Using TNBS

Mice were anesthetized using 3% isoflurane and sensitized with TNBS (Sigma-Aldrich, St Louis, MO, USA) by injecting 100 μl of a 1.0% TNBS solution in PBS subcutaneously one week prior to the administration of the TNBS rectal enema. Seven days post-sensitization, a lubricated 3.5F soft silicon catheter was inserted into the colon to a distance of 3–4 cm to inject a 50 μl enema containing 2.0 mg TNBS in a 50% ethanol/1X PBS solution. Following enema, mice were held inverted for 30 seconds before returning to the cage. Mice were weighed daily, and three days post-enema, mice were euthanized, colons removed, colon length measured, and colonic tissue prepared for histological analysis following fixation in 10% neutral buffered formalin. Hematoxylin/eosin staining was performed on formalin-fixed paraffin-embedded colonic sections and analyzed for inflammation, crypt hyperplasia, ulcerations, bowel wall thickening, and edema blinded for treatment group. Each histological parameter was scored 0 to 1 with a maximum score of 5.

### Immunohistochemistry

Slides were placed on the Leica Bond Max IHC stainer. All steps besides dehydration, clearing and cover-slipping are performed on the Bond Max. Slides were deparaffinized. Heat induced antigen retrieval was performed on the Bond Max using their Epitope Retrieval 2 solution for 20 min. The slides were incubated with anti-FOXP3 monoclonal antibody for 1 h at a 1:100 dilution followed by the visualization with Bond Polymer Refine detection system. Finally, slides were dehydrated, cleared and cover-slipped.

### Luminex

Simultaneous analysis of multiple cytokines and chemokines were assessed in plasma obtained from humanized mice using Luminex (MilliporeSigma, Burlington, MA, USA).

### Statistical Analysis

Statistical analysis was carried out using GraphPad Prism 8 software. *P* values were calculated using one-way analysis of variance (ANOVA) followed by Tukey’s multiple comparisons test and *P* < 0.05 was considered statistically significant.

## Results

### Treg Cell Expansion and Protection Against TNBS-Induced Colitis Is Independent of HLA-Restriction

**Figure 1:**
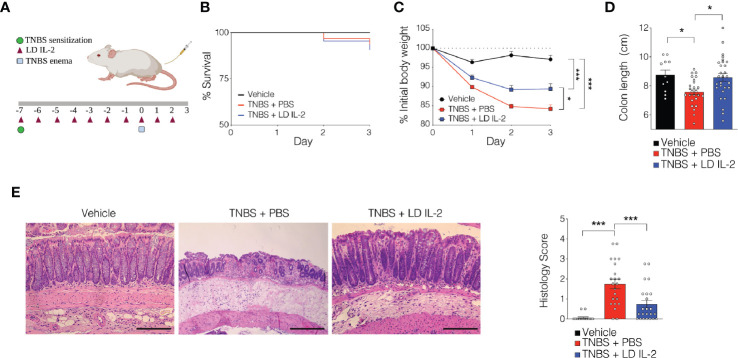
Low-dose (LD) interleukin-2 (IL-2)-mediated protection against trinitrobenzensulfonic acid (TNBS)-induced colitis is independent of T_reg_ cell human leukocyte antigen (HLA) restriction. **(A)** Schematic of LD IL-2/TNBS protocol. **(B)** Percent survival of NOD.*Prkdc^scid^Il2rg^-/-^* (NSG) human immune system (HIS) mice post-TNBS rectal administration (vehicle *n* = 11; PBS *n* = 28; LD IL-2 *n* = 30). **(C)** Percent initial body weight following induction of TNBS-induced colitis in NSG HIS mice treated with or without LD IL-2 (vehicle *n* = 11; PBS *n* = 28; LD IL-2 *n* = 30). **(D)** Colon length 3 days following vehicle or TNBS enema. Each dot represents an individual animal (vehicle *n* = 11; PBS *n* = 22; LD IL-2 *n* = 23). **(E)** Representative H&E-stained distal colon section at 10X magnification (left) and histology score (right) where each circle represents an individual animal (vehicle, *n* = 11; PBS *n* = 22; LD IL-2, *n* = 23). Scale bar = 200 µm. *P < 0.05, ***P < 0.001. Image generated using BioRender.

**Figure 2:**
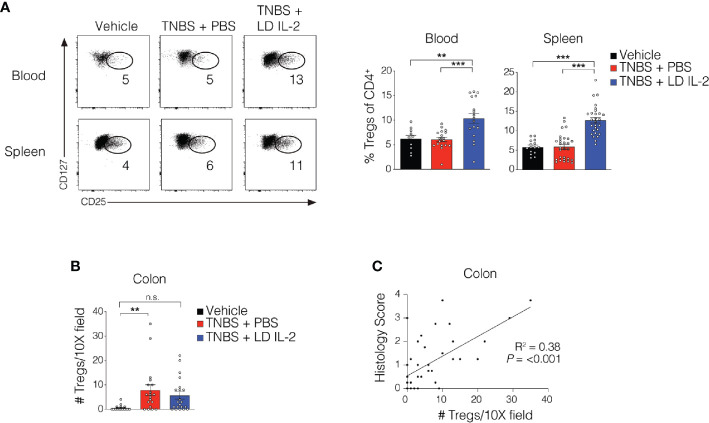
T_reg_ cell expansion in peripheral compartments following low-dose (LD) interleukin-2 (IL-2). **(A)** Representative flow cytometry dot plots of human T_reg_ cells of CD4^+^ in blood and spleen (left) with quantified data (right). **(B)** Quantification of FOXP3^+^ cells *via* immunohistochemistry in the colonic lamina propria. **(C)** Correlation between T_reg_ cell number and histology score in colon (R^2 =^ 0.38, *P* < 0.001). Data were pooled from three independent experiments and results were expressed as mean ± SEM. **P < 0.01, ***P < 0.001; n.s., not significant.

Recent work has demonstrated the efficacy of LD IL-2 as a therapeutic for several immune-mediated diseases (20–23). Previously, we reported that LD IL-2 may be an effective therapy to treat IBD based on our results whereby LD IL-2 induced human T_reg_ cell expansion and attenuated experimental colitis in immune humanized NSG mice expressing human HLA-DQ8 in a setting devoid of murine MHCII (28). To directly investigate the requirement of T_reg_ cell HLA restriction for LD IL-2-mediated protection, we employed standard immune humanized NSG mice purchased from The Jackson Laboratory. All mice were sensitized using a 1% TNBS solution injected subcutaneously. Mice were divided into 3 cohorts with one cohort receiving daily intraperitoneal injections of PBS and another with daily injections of 10,000 IU IL-2. After seven days, mice injected with PBS or LD IL-2 received a rectal challenge of TNBS in a 50% ethanol solution to induce colonic inflammation (**Figure 1A**). The third cohort that did not receive PBS or LD IL-2 was given an enema containing 50% ethanol as a vehicle control. Mice were weighted daily for three days. While there was no difference in mortality between the LD IL-2 treated or PBS treated groups (**Figure 1B**), humanized NSG mice treated with LD IL-2 exhibited accelerated recovery from TNBS-induced weight loss compared to mice treated with PBS as a control (*P* < 0.05) (**Figure 1C**). Colitis induction in mice often elicits a decrease in colon length that can be used as a prognosticator of inflammation. Therefore, we measured colon length at the experimental endpoint and found that LD IL-2 prevented TNBS-induced colonic shortening compared to PBS (*P* < 0.05) (**Figure 1D**). To determine the degree of inflammation microscopically, formalin-fixed paraffin-embedded (FFPE) distal colonic sections were stained with hematoxylin and eosin and scored in a blinded fashion. Although all mice receiving TNBS developed colitis, those receiving LD IL-2 exhibited reduced inflammation and injury compared to PBS (*P* < 0.001) (**Figure 1E**) that was similar to what we previously observed when CD4^+^ T cells were HLA restricted (28). As expected, NSG mice lacking human immune cells were not protected by LD IL-2 treatment (**Supplemental Figure 1**). Given the effect of LD IL-2 on T_reg_ cell expansion, we performed flow cytometric analysis on leukocytes isolated from the blood and spleen to determine T_reg_ cell frequency. LD IL-2 treatment increased T_reg_ frequencies in the blood and spleen of humanized mice compared to control and PBS treated mice (*P* < 0.01; *P* < 0.001) (**Figure 2A**). FFPE colon sections stained for FOXP3 showed a significant increase in the number of FOXP3^+^ cells in the colon of mice treated with PBS (*P* < 0.01) (**Figure 2B**). Interestingly, an increase in colonic FOXP3^+^ cells positively correlated with colitis severity (*P* < 0.001) (**Figure 2C**).

### LD IL-2 Treatment Modifies Select T Cell and NK Cell Subsets

**Figure 3:**
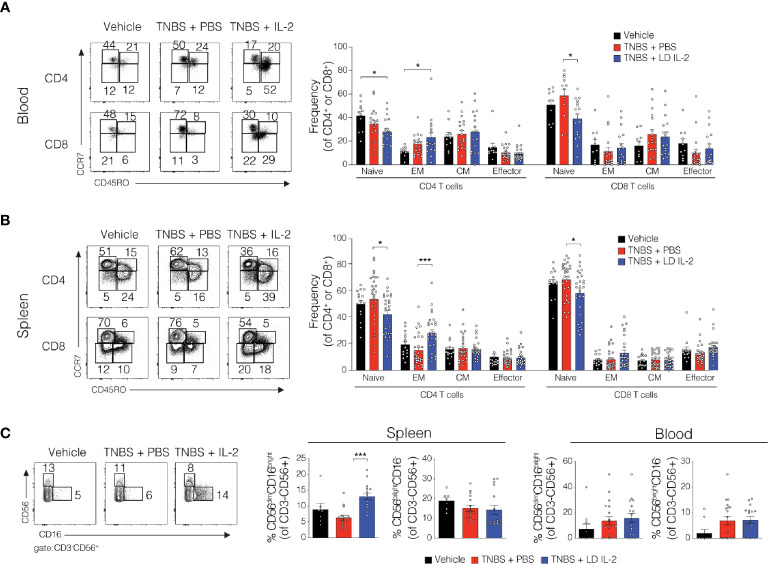
Alteration in the lymphocyte population in blood and spleen of trinitrobenzensulfonic acid (TNBS)-induced colitis in humanized NOD.*Prkdc^scid^Il2rg^-/-^* (NSG) mice treated with low-dose (LD) interleukin-2 (IL-2). **(A, B)** Representative flow cytometry dot plots and quantified data of human CD4^+^ and CD8^+^ T cells subsets in blood and *s*pleen where each circle represents an individual mouse. **(C)** Representative flow cytometry dot plots and quantified frequencies of splenic and blood natural killer (NK) cell subsets 3 days post-rectal challenge in NSG human immune system (HIS) mice (*n* ≥ 7). Data were pooled from three independent experiments and histograms depict the mean ± SEM. *P < 0.05, ***P < 0.001.

**Figure 4:**
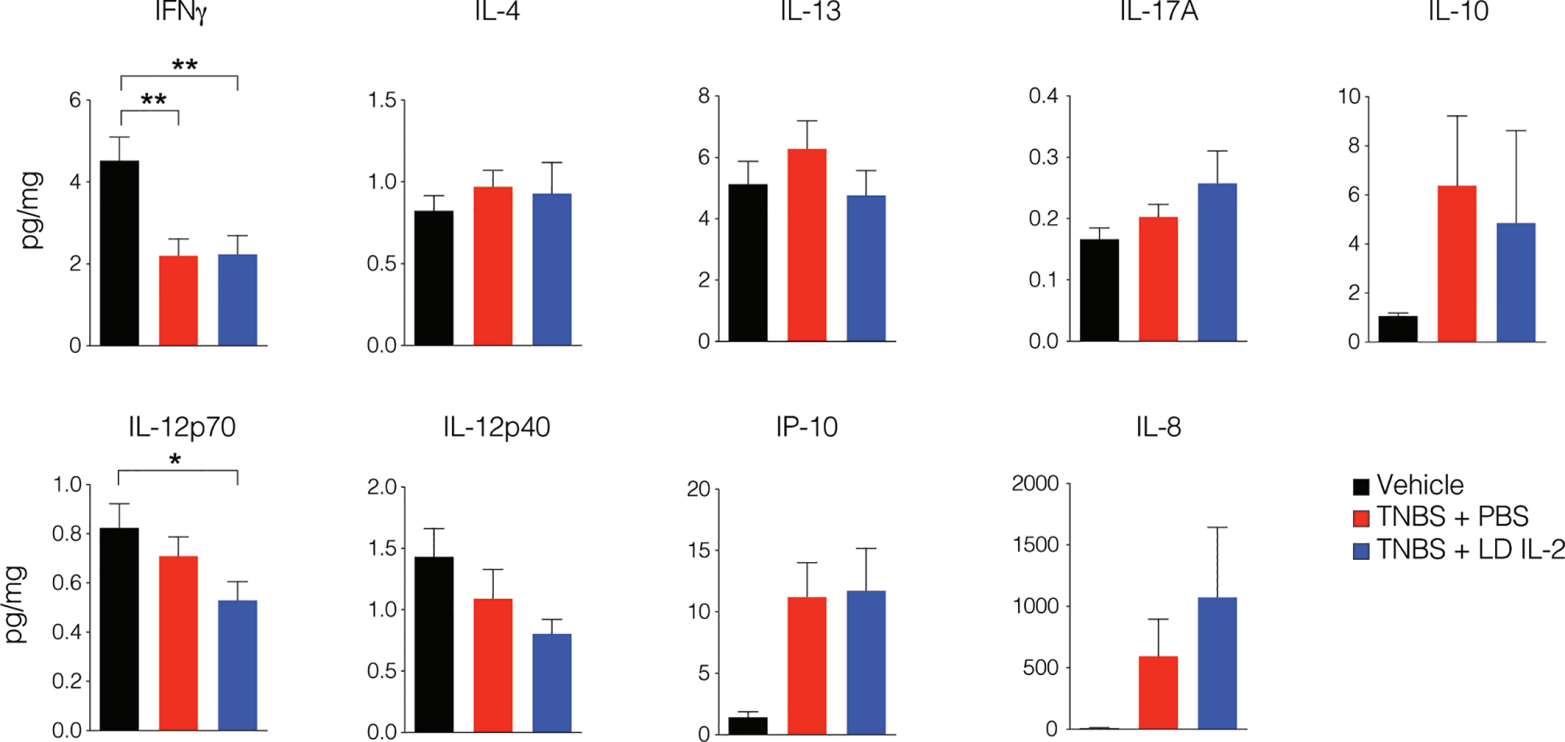
Interleukin-2 (IL-12) production is reduced in mice treated with low-dose (LD) interleukin-2 (IL-2). Concentration of human cytokines in colon tissue of NOD.*Prkdc^scid^Il2rg^-/-^* (NSG) human immune system (HIS) mice 3 days post-rectal challenge with trinitrobenzensulfonic acid (TNBS) or vehicle as a control determined by Luminex (*n* ≥ 11 per group). Histograms are the mean concentration pooled from three independent experiments ± SEM. *P < 0.05, **P < 0.01.

Conventional CD4^+^ and CD8^+^ T cells, as well as natural killer (NK) cells express the dimeric IL-2R (CD122 and IL-2Rγ) and are competent to bind IL-2, albeit with lower affinity. However, aberrant activation of conventional T or NK cells may elicit unwanted side effects. Therefore, we evaluated CD4^+^ and CD8^+^ T cell subsets for changes in frequency in the blood and spleen of humanized mice treated with TNBS treated with PBS or LD IL-2. We found a slight but statistically significant reduction in naïve CD4^+^ T cells (CCR7^+^CD45RO^-^) (*P* < 0.05) and an increase in effector memory CD4^+^ cells (CCR7^-^CD45RO^+^) in the blood with LD IL-2 treatment compared to PBS treated control, and a reduction in naïve CD8^+^ T cells following LD IL-2 treatment compared to PBS (*P* < 0.05) (**Figure 3A**). In the spleen, we observed a reduction in naïve CD4^+^ T cells and CD8^+^ T cells compared to PBS with an increased frequency of effector memory CD4^+^ similar to that observed in the blood (*P* < 0.05) (**Figure 3B**). Human NK cell subsets are often classified into two distinct subsets based on cell surface expression of CD56 and CD16 (35). The CD56^dim^CD16^+^ population of NK cells participates in the killing of target cells *via* perforin- and/or granzyme-mediated cytotoxicity (36) and we found a significant increase in this population following LD IL-2 treatment compared to PBS treated (*P* < 0.05) (**Figure 3C**). Interestingly, the CD56^bright^CD16^dim^ population of NK cells is described as being immunoregulatory and can proliferate in response to IL-2 (37), yet this population was not altered following LD IL-2 treatment (**Figure 3C**). Collectively, these data indicate that the majority of CD4^+^ and CD8^+^ T cell subsets are not significantly altered by LD IL-2, whereas CD56^dim^CD16^bright^ NK cells displayed increased frequency following treatment with LD IL-2. We also quantified several human cytokines in distal colon tissue and while LD IL-2 reduced IL-12 production in colon tissue of TNBS-treated mice compared to vehicle treated control (**Figure 4**), the majority of cytokines evaluated were not significantly altered, suggesting that LD IL-2 may be acting on T_reg_ cells in non-colon tissue.

## Discussion

Here we show that LD IL-2 treatment of immune humanized NSG mice induced human T_reg_ cell expansion and was protective against experimental colitis and adds to our previous findings using HLA-restricted humanized mice (28). Interestingly, while the expansion of T_reg_ cells in the periphery inversely correlated with disease severity, increased T_reg_ cell numbers in the colonic lamina propria positively correlated with disease activity. Given that effector CD4^+^ T cells in humans can upregulate FOXP3 upon activation (38, 39), the true identity of these cells is presently unclear. Importantly, these data show that LD IL-2-induced T_reg_ expansion and protection against TNBS-induced colitis was independent of T_reg_ cell HLA-restriction. The significance of these findings are that, while some disease models require T cell HLA-restriction in HIS mice (26), evaluation of IBD therapeutics directed at human T_reg_ cell expansion and/or function does not require T cell HLA-restriction to attenuate TNBS-induced colitis.

Immunodeficient mice harboring a human immune system are a valuable tractable model for a variety of pre-clinical research applications including leukemia, HIV, and immuno-oncology. Moreover, the ability to directly assess human therapeutic targets *in vivo* has the potential to inform novel clinical approaches. In recent years, LD IL-2 has been recognized as an effective therapeutic for treating patients with chronic inflammatory disorders (20–23). Previously we and others showed a requirement for human CD4 T cell selection on human HLA to generate appropriate adaptive immune responses in humanized mice (26, 40). Reasoning that T cell activation is a component of inflammation in the intestine, we initially employed NSG mice expressing human HLA to assess LD IL-2 as a therapeutic to expand human T_reg_ cells and suppress experimental colitis (27, 28). While our current study highlights the therapeutic potential for LD IL-2 in treating patients with IBD, one limitation is that colitis induction by TNBS does not require human cells, which is in contrast to what we had previously observed (27). It is likely that the use of ketamine/xylazine, and accompanying extended duration of anesthetization, in the initial study necessitated a lower TNBS dose that had modest effects on non-reconstituted mice (27). In this study, the rapid recovery from 3% isoflurane as an anesthetic required a higher TNBS dose to induce similar degrees of colonic inflammation that did not require the presence of human cells. Thus, non-humanized NSG mice are also susceptible to TNBS-induced inflammation. Nevertheless, the therapeutic effect of LD IL-2 was dependent on the presence of human immune cells. Whether expanded human T_reg_ cells are able to restrict inflammation through direct actions on human immune cells or limit recruitment/activation of murine innate immune cells is not entirely clear. T_reg_ cells are known to suppress immune responses through both contact-dependent and -independent mechanisms (41, 42) and murine cells are responsive to many human cytokines.

For patients with IBD, the number of FOXP3^+^ cells is often increased within inflammatory lesions (43). Yet, these potent immunoregulatory cells are unable to restore immune homeostasis (44–47). While it is possible that there are still too few regulatory cells to restrain exacerbated inflammatory responses, restricting effector cells in peripheral compartments may play a more significant role in limiting inflammation at least in acute settings. This would be consistent with our data showing peripheral LD IL-2-mediated T_reg_ cell expansion was protective. Conversely, increased FOXP3^+^ cells in colon tissue correlated with increased disease activity. This suggests that T_reg_ cell expansion and suppression of colitis in this model, and perhaps in humans as well, may by more critical in peripheral compartments rather than at the site of inflammation. The increased sensitivity of T_reg_ cells for IL-2 compared to conventional T cells is an important property to maintain selectivity. Importantly, we did not observe expansion of conventional CD4^+^ or CD8^+^ T cells in the blood or spleen with LD IL-2 treatment. We did, however, observe an increase in splenic CD56^dim^CD16^+^ NK cells, a population which has been described as being cytolytic, while CD56^bright^CD16^-^ NK cells were not altered. This finding was unexpected given that CD56^bright^ NK cells are reported to express the high affinity IL-2R and are competent to respond to lower concentrations of IL-2 (48). It has also been reported that NK cells exposed to an immunoregulatory environment can also lower CD56 expression (49), which may be expected in the setting of LD IL-2 given the expansion of regulatory cells and should be considered in future studies and applications.

Collectively, our findings support the therapeutic use of LD IL-2 to expand human T_reg_ cells to treat patients with IBD. Based on our model, it appears unlikely that LD IL-2-mediated T_reg_ expansion and suppression of immune responses occur in an antigen-specific manner, given that LD IL-2 therapeutic efficacy was observed in humanized NSG mice lacking HLA molecules and therefore limit T_reg_ cell TCR-HLA interactions. Thus, immune humanized NSG mice are an ideal system in which to evaluate novel therapeutic strategies targeting human T_reg_ cell expansion and/or function in conjunction with experimental colitis as a readout for therapeutic efficacy. Broad commercial availability of immune humanized NSG mice presents a low hurdle that we now demonstrate enables rapid evaluation and innovative approaches aimed at modulating human T_reg_ cells for clinical applications.

## Data Availability Statement

The raw data supporting the conclusions of this article will be made available by the authors, without reservation.

## Ethics Statement

The animal study was reviewed and approved by The Institutional Animal Care and Use Committee at Vanderbilt University Medical Center.

## Author Contributions

RT, JJ, JL, ES, KO, and JG conceived and designed the study. RT, JJ, JL, and JG performed humanized mouse experiments. RT, JL, JJ, ES, MA, KW, KO, and JG analyzed and interpreted data. RT, JJ, and JG wrote the manuscript. All authors contributed to the article and approved the submitted version.

## Funding

The work was supported by grants from the National Institute of Diabetes and Digestive and Kidney Diseases DK106311, Crohn’s and Colitis Foundation CDA 352644 (JG). Pilot Grant from The Vanderbilt Digestive Disease Research Center P30 DK058404. The Academy Ter Meulen Grant from the Royal Netherlands Academy of Arts and Sciences and the Cultural Foundation Grant from the Prince Bernhard Cultural Foundation (to JJ). Crohn's & Colitis Foundation Senior Research Award #703003 (KTW).

## Conflict of Interest

ES and KO are employed by Pandion Therapeutics.

The authors declare that financial support for some of the humanized murine studies was provided by Pandion Therapeutics. The funder was, in part, involved in study design and procurement of mice. The data collection and analysis was not a part of the funding provided. The authors shared the results of the study with Pandion and shared the manuscript draft for critiques prior to submission.

The remaining authors declare that the research was conducted in the absence of any commercial or financial relationships that could be construed as a potential conflict of interest.

## Supplementary Material

Supplementary Figure 1 LD IL-2-mediated protection against TNBS colitis requires human immune cells. Weight loss following TNBS enema in NSG mice devoid of human cells treated with (*n* = 7) or without (*n* = 6) LD IL-2.
