# Supplementary figures and images for: HLA-Restriction of Human Treg Cells Is Not Required for Therapeutic Efficacy of Low-Dose IL-2 in Humanized Mice
Source: Front Immunol. 2021 Feb 24;12:630204. doi: 10.3389/fimmu.2021.630204 (PMC7945590; doi:10.3389/fimmu.2021.630204)

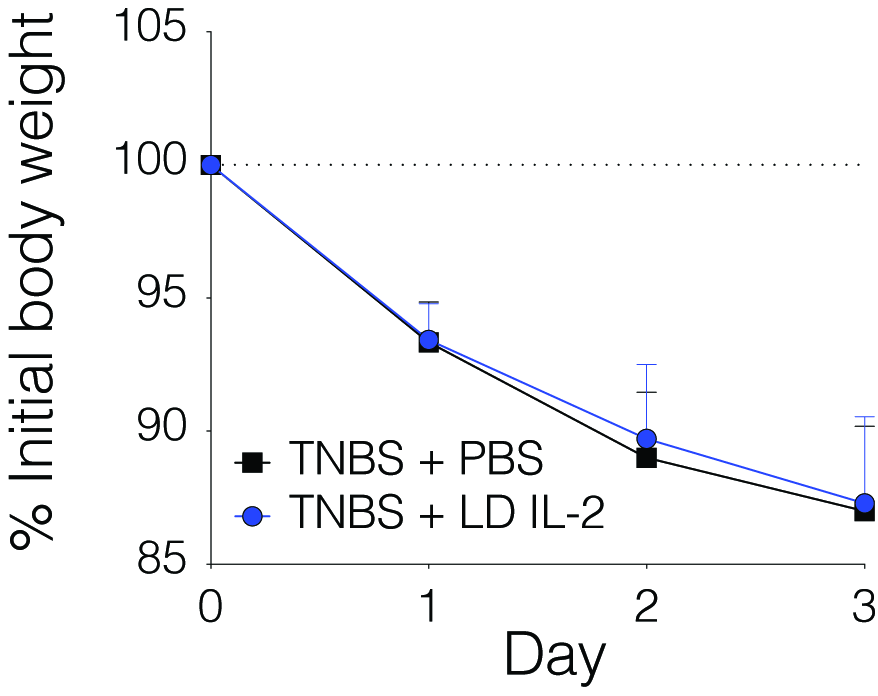

Supplement: Supplementary Figure 1 — LD IL-2-mediated protection against TNBS colitis requires human immune cells. Weight loss following TNBS enema in NSG mice devoid of human cells treated with (n = 7) or without (n = 6) LD IL-2. [file Image_1.tif]
